# Novel missense mutation in the bZIP transcription factor, *MAF*, associated with congenital cataract, developmental delay, seizures and hearing loss (Aymé-Gripp syndrome)

**DOI:** 10.1186/s12881-017-0414-7

**Published:** 2017-05-08

**Authors:** Shari Javadiyan, Jamie E. Craig, Shiwani Sharma, Karen M. Lower, Theresa Casey, Eric Haan, Emmanuelle Souzeau, Kathryn P. Burdon

**Affiliations:** 1 Department of Ophthalmology, School of Medicine, Flinders University, Adelaide, Australia; 2 Department of Haematology and Genetic Pathology, School of Medicine, Flinders University, Adelaide, Australia; 3 Ophthalmology Department, Women’s and Children’s Hospital, Adelaide, Australia; 4 SA Clinical Genetics Service, SA Pathology (at Women’s and Children’s Hospital), Adelaide, Australia; 5 School of Medicine, University of Adelaide, Adelaide, Australia; 6 Menzies Institute for Medical Research, University of Tasmania, Hobart, Australia

**Keywords:** *MAF*, Congenital cataract, Pediatric cataract, Ion Ampliseq, Next generation sequencing, Syndromic cataract, Aymé-Gripp syndrome

## Abstract

**Background:**

Cataract is a major cause of severe visual impairment in childhood. The purpose of this study was to determine the genetic cause of syndromic congenital cataract in an Australian mother and son.

**Method:**

Fifty-one genes associated with congenital cataract were sequenced in the proband using a custom Ampliseq library on the Ion Torrent Personal Genome Machine (PGM). Reads were aligned against the human genome (hg19) and variants were annotated. Variants were prioritised for validation by Sanger sequencing if they were novel, rare or previously reported to be associated with paediatric cataract and were predicted to be protein changing. Variants were assessed for segregation with the phenotype in the affected mother.

**Result:**

A novel likely pathogenic variant was identified in the transactivation domain of the *MAF* gene (c.176C > G, p.(Pro59Arg)) in the proband and his affected mother., but was absent in 326 unrelated controls and absent from public variant databases.

**Conclusion:**

The *MAF* variant is the likely cause of the congenital cataract, Asperger syndrome, seizures, hearing loss and facial characteristics in the proband, providinga diagnosis of Aymé-Gripp syndrome for the family.

**Electronic supplementary material:**

The online version of this article (doi:10.1186/s12881-017-0414-7) contains supplementary material, which is available to authorized users.

## Background

Cataract is an opacity of the crystalline lens resulting in impaired vision. Cataract formation is typically an age-related process that affects adults; however, rarely it can be present at birth or early childhood and is classified as congenital (or juvenile/paediatric) cataract. Congenital cataract occurs in 1–6 per 10,000 live births in developed countries [1]; in Australia the incidence is estimated to be 2.2 per 10,000 births [2].

Around 50% of cases have a genetic cause [3], with other causes including intrauterine infection, malnutrition and metabolic disorder. Hereditary congenital cataracts can be transmitted as autosomal recessive, autosomal dominant or X-linked traits, with autosomal dominant the most common mode of inheritance, and can be isolated or syndromic (associated with additional non-ocular abnormalities) [4]. The disorder demonstrates genetic and phenotypic heterogeneity.

Among the many genes associated with congenital cataracts is the transcription factor gene *MAF* (*v-maf avian musculoaponeurotic fibrosarcoma oncogene homolog*, OMIM 177075, NM_005360.4). The MAF family of transcription factors is divided into two subgroups, large and small. The large subgroup (*MAFA*, *MAFB*, *c-MAF* or *v-MAF*, and retina-specific leucine zipper (*NRL*)) is characterized by a bZip structure, a motif for DNA binding and protein dimerization and a transactivation domain [5]. The small MAF proteins (MAFF, MAFG, and MAFK) lack the transactivation domain [5, 6].

Here we report a missense variant in *v-MAF*, usually referred to as *MAF*, in a mother and son with syndromic congenital cataract associated with hearing loss and developmental delay.

## Methods

DNA was extracted from whole blood using the QIAamp DNA blood maxi kit (Qiagen, Hilden, Germany) according to the manufacturer’s protocol. Fifty-one genes associated with congenital cataract human or mouse were selected through a review of the literature (Additional file 1: Table S1) [7–20] for sequencing the coding and untranslated regions.

**Fig. 1:**
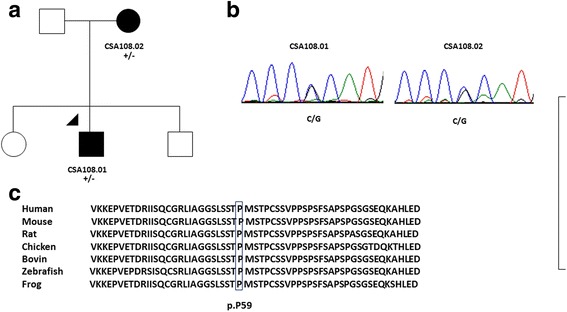
**a** Pedigree of family CSA108 with variant in *MAF*. Individuals with ID numbers were examined by an ophthalmologist. *Solid circles* indicate affected females and *solid squares* indicate affected males. The proband is marked by an *arrow head*. “+” indicates mutant allele and “−” indicates wild type allele of c.176C > G in the *MAF* gene. **b** Sequence chromatogram of two examined individuals at variant c.176C > G. Both sequenced affected members are heterozygous for this variant. **c** Protein alignment shows the MAF protein is highly conserved among the indicated species. The mutated residue is indicated by the *box*

A sequencing library was prepared using the proband’s DNA (patient CSA108.01, Fig. 1a) as template. The library was generated with the Ion AmpliSeq library kit version 2.0 (Life Technologies, California, USA) and custom Ion Ampliseq primers according to the manufacturer’s protocols, and was sequenced on an Ion Torrent Personal Genome Machine using the Ion PGM Sequencing 200 Kit v2 and an Ion 318 chip (Life Technologies). Alignment to the reference genome (hg19), variant calling and annotation were conducted in Torrent Suite (version 3.6.), and Ion reporter (V4.0) with appropriate plugins. Variants were prioritized for further analysis if they were predicted to alter protein sequence (non-synonymous), were absent or very rare (Minor Allele Frequency < 1%) in public databases including dbSNP137 (https://www.ncbi.nlm.nih.gov/SNP/), Exome Aggregation Consortium (ExAC) (http://exac.broadinstitute.org/) and absent from an in-house list of sequencing errors previously seen on this gene panel. In addition, selected variants were assessed by SIFT [21] and Polyphen-2 [22] for their predicted effect on protein function.

The detected novel, coding variant in *MAF* was validated by Sanger sequencing using forward primer 5′-GGGGGTGTGTGTGTGAGC-3′ and reverse primer 5′-CTGGAGCTGGTGGCTGTT-3′. PCR reactions of 20 μl final volume consisting of 1X Coraload PCR buffer (Qiagen), 0.1 mM dNTPs (Roche Diagnostics, Basel, Switzerland), 0.5 μM each primer, 0.5U Hot Star Plus Taq Polymerase (Qiagen) and 40 ng of DNA was prepared. Final concentrating of Mg^2+^ was adjusted to 2.5 mM by adding the required amount of Mgcl_2_ (Qiagen). Amplification conditions involved an initial activation step of 95 °C for 5 min, followed by 35 cycles of 30 s of denaturation at 95 °C, 30 s of annealing at 57 °C and 30 s of extension at 72 °C. A final extension step was for 5 min at 72 °C. The PCR products were cleaned by treatment with 10 units (U) Exonuclease I (New England Biolabs, Genesearch Pty Ltd, QLD, Australia) and 2 U of Shrimp Alkaline Phosphatase (SAP) (USB, Millennium Science Pty. Ltd., VIC, Australia) at 37 °C for 1 h followed by enzyme deactivation at 80 °C for 20 min.

The cleaned PCR product was sequenced with BigDye Terminators (Life Technologies) on an ABI3300xl according to standard protocols. The variant was screened in 326 unrelated normal Australian controls using the MassArray platform (Sequenom, California, USA) and iPlEX chemistry (Sequenom) at the Australian Genome Research Facility (QLD, Australia) and assessed for conservation across species using The Universal Protein Resource (UniProt) database (http://www.uniprot.org/).

## Results

DNA from the proband was sequenced for 51 known congenital cataract genes using an Ion AmpliSeq custom amplicon panel. A total of 1,023,730 reads were mapped against the reference genome (hg19), of which 94.18% were on target. An average read depth of 841.9× was achieved for a total of 1216 amplicons with 96.05% of the target bases covered at least 20 fold. A total of 134 variants were annotated (Additional file 2), of which only six were novel or rare and nonsynonymous. Of the six variants, only one was not present in an in-house list of sequencing errors previously seen on this gene panel and predicted to be pathogenic by both SIFT and Polyphen-2 (five variants were false positive). This novel coding variant was a missense variant in the *MAF* gene (c.176C > G, p.(Pro59Arg)) (Fig. 1b). It was predicted to be pathogenic by SIFT and Polyphen-2 and is in a highly conserved region of the protein (Fig. 1c). The variant was also present in the affected mother and absent in 326 screened unrelated Caucasian controls. DNA was not available from the two unaffected siblings.

**Fig. 2:**
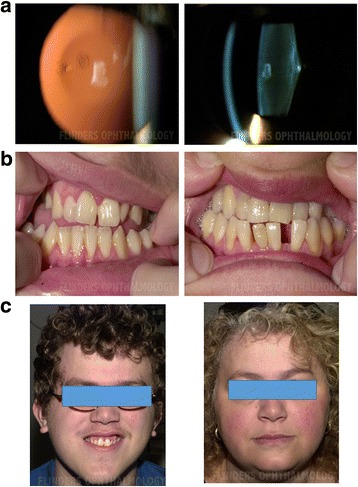
Clinical features of the syndrome in family CSA108. **a** Phenotype of syndromic cataract in CSA108.01. Slit-lamp photographs showing posterior polar oil droplet cataract with posterior lenticonus. **b** Dental abnormalities in CSA108.01 (*left*) and CSA108.02 (*right*). **c** Facial features in CSA108.01 (*left*) and CSA108.02 (*right*). In particular, note flat mid-face in both, and short philtrum, long/narrow chin and upturned ear lobules in CSA108.01

The 20-year-old proband (CS108.01) was diagnosed at birth with bilateral congenital cataract, described as nuclear and posterior polar in the right eye, and milder posterior polar oil droplet cataract in the left eye (Fig. 2a). Cataract in the right eye was removed at 5 months of age and the patient subsequently developed aphakic glaucoma. The right eye ultimately was significantly amblyopic. The proband also had mild to moderate sensorineural hearing loss (he did not appear to have a hearing impairment in early childhood). He was diagnosed with Asperger syndrome and borderline intellectual abilities in childhood. He attended a special school because of the combination of Asperger syndrome and visual/hearing impairment. In spite of this, he completed secondary education and went on to university, implying normal intellectual abilities. His childhood assessments of mild intellectual disability and borderline abilities are likely to have reflected the autism spectrum disorder and possibly the visual impairment. He developed scoliosis during teenage and had seizures at 13.5 years (two, 2 weeks apart). His height was 25th–50th percentile and head circumference was 50th–98th percentile. He had a distinctive facial appearance with narrow posteriorly rotated ears with upturned ear lobules, downslanting palpebral fissures, flat mid-face, short philtrum, prominent narrow chin and dental malocclusion (Fig. 2c). There was no joint limitation. The 53-year-old mother of the proband, CSA108.02, who had a mild learning disability and hearing impairment, had cataract extraction at the age of 40. She had not been diagnosed to have an autism spectrum disorder, had not had seizures and did not have joint limitation. Other features were normal height (10th–25th percentile), premature hair loss and double nails, with a nail growing out over the top of the existing one. She had mildly down slanting palpebral fissures, flat mid-face, a relatively prominent chin and widely spaced lower teeth (Fig. 2b).

## Discussion

Undergoing traditional diagnostic assessment procedures, the clinical diagnosis of this proband with a rare syndromic congenital cataract phenotype was complicated and protracted. Past investigations included brain MRI, EEG, karyotyping, subtelomere FISH, FISH for Smith-Magenis syndrome, TORCH serology, urine metabolic screen for amino acids, organic acids and mucopolysaccharides, galactose-1-phosphate uridyl transferase, 7-dehydrocholesterol and very long chain fatty acids; all were normal apart from a diffusely abnormal EEG. Sequencing of the *NHS* gene associated with Nance-Horan syndrome (congenital cataract, dental anomalies and developmental delay) did not detect any pathogenic variants. The implementation of next generation sequencing including targeted gene sequencing panels such as PGM (Personal Genome Machine) results in more convenient molecular diagnostic process.

**Fig. 3:**
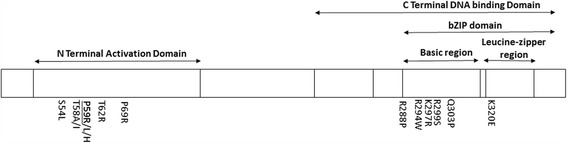
Schematic of the human MAF protein indicating the positions of reported variants (Adapted from Niceta et al. [27]). The protein contains an N-terminal transactivation domain and a C-terminal DNA binding domain. The C-terminal domain consists of an extended homology region, basic region (aa288–313) and leucine-zipper region (aa316–aa337). The variants associated with Aymé-Gripp syndrome are located in the N-terminal transactivation domain including the variant (p.(Pro59Arg)) reported here (*bolded* and *underlined*). Other variants are located within the C-terminal DNA-binding domain and are associated with other forms of congenital cataract mainly isolated

Although this study cannot entirely rule out a novel cause for disease, the features described in the proband and his mother are consistent with the condition previously reported independently by Aymé and Phillip [23] and Gripp et al. [24] (MIM 601088). There also have been reports of a similar syndrome by Fine and Lubinsky [25] and Preus et al. [26]. A recent study by Niceta et al. [27] reported a narrow spectrum of amino-acid substitutions within the MAF protein (Fig. 3), causing cataract, deafness, intellectual disability, seizures, a distinctive flat facial appearance, skeletal anomalies and reduced growth. The authors proposed the eponym Aymé-Gripp for this multisystem disorder. The reported *de novo* amino acid substitutions in *MAF* associated with this syndrome are p.(Ser54Leu), p.(Thr58Ala), p.(Thr58Ile), p.(Pro59His), p.(Pro59Leu), p.(Thr2Arg) and p.(Pro69Arg). Interestingly all these variants are located within the N-terminal transactivation domain of *MAF* as is the p.(Pro59Arg) substitution reported here (Fig. 3). Unlike other reported variants in *MAF* associated with Aymé-Gripp syndrome [27], the variant described here was inherited, with transmission from mother to son. Our findings also show that variants associated with Aymé-Gripp syndrome can display intra-familial variability since the mother had a substantially milder phenotype than the proband.

There also have been multiple other reports of variants in *MAF* associated with various forms of congenital cataract (Fig. 3): p.(Arg294Trp), p.(Lys297Arg), p.(Arg299Ser) and p.(Lys320Gly) variants have been linked with nuclear congenital cataract, [28] cerulean congenital cataract and microcornea [29], lamellar cataract with microcornea and iris coloboma, [30] and nuclear, punctate, stromal cataract with microcornea [3], respectively. Jamieson et al. described a family with juvenile onset progressive cataract of cortical pulverulent opacities with anterior and posterior sutural densities, anterior segment dysgenesis and microphthalmia associated with the cytogenetically balanced chromosome translocation 46, XY, t(5;16) (p15.3;q23.2), which transected the genomic-control domain of *MAF* [31]. They also reported a variant in the DNA-binding domain of *MAF* (p.(Arg288Pro)) in a three generations family with lamellar cortical and nuclear pulverulent cataract, microcornea, and iris coloboma. Narumi et al. [32] identified a *MAF* variant (p.(Gln303Leu)) through whole exome sequencing in a family with phenotypically variable congenital cataract (lamellar or anterior polar with microcornea and iris coloboma). The affected proband was diagnosed with lamellar cataract without any other eye malformation with language development delay and autism. The proband was also screened for variants in the *NHS* gene, however similar to the case we are reporting here, no variant in this gene was detected.

Many transcription factor genes are involved in lens development and their functions are important for proper lens induction, and cell proliferation and differentiation [33]. MAF belongs to the bZIP transcription factor family. It forms both homodimers and heterodimers, and binds to MAF response elements in target genes [7]. *MAF* is expressed in lens fibre cells during lens development and it has been demonstrated that homozygous *Maf* mutant mice had defective differentiation of lens fibre cells [34]. MAF has been proposed to regulate the expression of the lens specific genes including *Crystallins* [33, 35]. It has been demonstrated that the activity of the large MAF transcription factors is strongly dependent on phosphorylation within the conserved transactivation domain of the protein. This domain is rich in aspartic acid, glutamic acid, serine, threonine and proline residues [5, 6].

MAF is phosphorylated by glycogen synthase kinase 3 (GSK3), a serine/threonine protein kinase. Phosphorylation increases the transactivation activity of MAF and induces protein degradation [36]. GSK3 requires a priming phosphorylation on the substrate four amino acids C-terminal of the target phosphorylation sites [27, 37]. It has been demonstrated that the Thr58 residue is one of the GSK3 phosphorylation target sites [36] and residue Pro59 is located between the target (Thr58) and primed residues (Thr62) and is essential for GSK3 phosphorylation activity. Thus, substitution of this residue to Arginine, as seen in this family, might impair GSK3-mediated phosphorylation of MAF proteins, resulting in inefficient ubiquitination of the transcription factor and its decreased degradation and functional dysregulation. Consistently, this residue is highly conserved between species including mammals, birds, fish and amphibians, further suggesting its functional importance. Other variants at the same residue p.(Pro59His) and p.(Pro59Leu) [27] have been also reported to cause a similar phenotype.

## Conclusion

We report a case of syndromic congenital cataract with similar features to those described by Niceta et al [27]. Ayme-Gripp syndrome’s key features are congenital cataracts, sensorineural hearing loss, intellectual disability, seizures, brachycephaly, flat face and short stature. The proband displayed all of the features except intellectual disability (though he has Asperger syndrome) and short stature. These features, combined with the novel identified variant in the transactivation domain of *MAF*, are consistent with a diagnosis of Aymé-Gripp syndrome in this family. This case is one of the most mildly affected reported and this is the first report of an inherited variant associated with this syndrome. This study shows the power and feasibility of next generation sequencing for variant detection in a clinically and genetically heterogeneous condition like syndromic congenital cataract, and demonstrates the ability of the technology to diagnose patients on the basis of their genetic results in combination with their phenotypic data.

## Additional files

Additional file 1: Table S1. List of reported paediatric cataract genes selected for sequencing in this study. (DOCX 39 kb)

Additional file 2: List of  annotated and selected variants. (XLSX 41 kb)

## Abbreviations

ExAC: Exome Aggregation Consortium
MAF: *v-maf avian musculoaponeurotic fibrosarcoma oncogene homolog*
PCR: Polymerase Chain Reaction
PGM: Personal Genome Machine
